# Supplementary material for: Incidence of Anorexia Nervosa in Women: A Systematic Review and Meta-Analysis
Source: Int J Environ Res Public Health. 2020 May 28;17(11):3824. doi: 10.3390/ijerph17113824 (PMC7312606; doi:10.3390/ijerph17113824)
Supplement: Supplementary file 1 [file ijerph-17-03824-s001.pdf]

Table S1. Database literature searches from 1980 until December 2019.

**Database**	**Search stategy**
Embase	('anorexia nervosa'/exp OR 'anorexia nervosa' OR (('anorexia'/exp OR anorexia) AND nervosa)) AND ('incidence'/exp OR incidence)
Pubmed	"incidence" [MeSH Terms] AND "anorexia nervosa" [MeSH Terms] and female
Web of Science	(Incidence and anorexia nervosa), (Anorexia nervosa women) and (Anorexia nervosa epidemiology)
Scopus	(“incidence” AND “anorexia nervosa” AND “women”) AND pubyear <1979 AND pubyear <2020

Table S2. Quality of the papers included in the systematic review by The Newcastle-Ottawa scale.

Authors	Representativeness	Sample size	Non-responders	Exposure	Comparability	Outcome	Statistical test	Global
Kendell et al. 1973	**1**	**1**	**1**	**0**	**0**	**1**	**0**	**4**
Jones et at. 1980	1	0	0	0	0	2	0	3
Hoek & Brook, 1985	0	0	0	2	1	2	0	5
Szmukler, 1986	1	1	1	2	1	1	0	7
Cullberg & Engstrom-Lindberg, 1988	1	0	0	2	0	0	0	3
Nielsen, 1990	1	0	0	2	2	2	1	8
Willi & Grossmann, 1983	1	1	1	1	1	1	0	7
Joergensen, 1992	1	0	0	2	0	2	0	5
Møller-Madsen & Nystrup, 1992	1	1	1	2	1	2	0	8
Pagsberg & Wang, 1994	1	0	0	2	1	2	0	6
Munk-Jøergensen et al. 1995	1	0	1	2	1	2	0	7
Rooney et al. 1995	1	1	0	2	0	1	0	5
Turnbull et al. 1996	1	0	0	2	1	2	1	7
Lucas et al. 1999	1	0	0	2	1	2	0	6
Ghaderi & Scott, 2001	1	0	0	2	2	1	1	7
Milos et al. 2004	1	0	0	2	1	2	1	7
Lahortiga-Ramos et al. 2004	1	0	0	2	1	1	1	6
Hoek et al. 2005	0	0	0	2	0	2	1	5
Currin et al. 2005	1	0	0	2	1	2	0	6
van Son et al. 2006	1	1	1	2	1	2	1	9
Keski-Rahkonen et al. 2007	1	1	0	2	1	1	1	7
Sigurdardotti et al. 2010	1	1	1	2	1	1	0	7
Pavlova et al. 2010	1	1	1	2	1	2	1	9
Micali et al.2014	1	0	0	2	1	2	1	7
Steinhausen & Jensen, 2015	1	1	0	2	1	2	0	7
Holland et al. 2016	1	1	0	2	1	2	1	8
Zerwas et al. 2015	1	0	0	2	1	2	1	7
Smink et al. 2016	1	1	0	2	1	1	1	7
Tsai et al. 2018	1	1	1	2	1	2	1	9
Reas & Rø, 2018	1	1	1	2	1	2	1	9
Mei-Chih et al. 2019	1	1	0	2	1	2	1	9

Table S3. Incidence of anorexia nervosa per 100,000 woman-years in hospital admissions.

Study	Country	Criteria	Ages	Period	N	n	Incidence	95% CI
Jones,1980 [27]	USA	No data	All	1960-69	3265306	16	0.5	0.28- 0.80
				1970-76	2416666	29	1.2[†]	0.80-1.72
			15-24	1960-69	545454	3	0.6	0.13-1.61
				1970-76	460122	15	3.3	1.82-5.38
Hoek & Brook, 1985 [29]	Netherlands	ICD-9	15-24	1974-82	22500	84	37.1	29.80-46.22
Szmukler, 1986 [35]	Scotland	ICD-8	16-25	1978-82	207932	6	3.0[η]	1.06-6.28
Nielsen, 1990 [30]	Denmark	ICD-8	15-24	1973-87	8400	57	6.8	5.14-8.79
Willi & Grossman 1983 [32]	Switzerland	Feighner/DSM-III	12-25	1956-58	250000	10	4.0	1.92-7.36
				1963-65	250000	17	6.8	3.96-10.89
				1973-75	226190	38	16.8	11.89-23.06
Joergensen, 1992 [31]	USA	ICD-8	10-25	1977-86	5600	62	11.0	8.49-14.19
Møller-Madsen & Nystrup, 1992 [34]	Denmark	ICD-8	15-24	1970	359344[ϑ]	12	3.4	1.73-5.83
				1987		43	12.0	8.66-16.12
				1989		32	9.0	6.09-12.57
Munk-Jøergensen, 1995 [28]	Denmark	ICD-8[¶]	10-29	1970-93	9982420[†]	956	9.6	8.98-10.20
			All	1970-93	63196800	1100	1.7	1.64-1.85
Milos et al. 2004 [33]	Switzerland	DSM-III-R	12-25	1983-85	291900	48	16.4	12.12-21.80
				1993-95	207900	41	19.7	14.15-26.75
Sigurdardottir, 2010 [6]	Iceland	ICD-9/ICD-10	11-46	1983-08	1837209	79	4.3	3.40-5.30
Pavlova et al. 2010 [26]	Czech Republic	ICD-10	10-39	1994	5151604	233	4.5[γ]	3.96-5.14
				1995		307	6.0[γ]	5.31-6.66
				1996		298	5.8[γ]	5.15-6.48
				1997		278	5.4[γ]	4.78-6.07

				1998		330	6.4[γ]	5.73-7.14
				1999		385	7.5[γ]	6.75-8.26
				2000		359	7.0[γ]	6.27-7.73
				2001		297	5.8[γ]	5.13-6.46
				2002	5117046	314	6.1[γ]	5.48-6.85
				2003		319	6.2[γ]	5.57-6.96
				2004		321	6.2[γ]	5.61-7.00
				2005		326	6.4[γ]	5.57-6.70
Holland et al. 2016 [25]	England	ICD-8	10-44	1968-71[δ]	851852	23	2.7	1.71-4.05
				1972-76[δ]	1700000	51	3.0	2.23-3.94
				1977-81[δ]	2757574	91	3.3	2.66-4.05
		ICD-9		1982-86[δ]	2920000	73	2.5	1.96-3.14
				1987-91[δ]	2863636	63	2.2	1.69-2.81
				1992-96[δ]	3074074	83	2.7	2.15-3.35
		ICD-10		1997-01[δ]	2822222	127	4.5	3.74-5.35
				2002-06[δ]	3183673	156	4.9	4.16-5.73
				2007-11[δ]	3285714	207	6.3	5.47-7.22
				1997-01[ε]	33238095	1396	4.2	3.98-4.43
				2002-06[ε]	58431818	2571	4.4	4.23-4.57
				2007-11[ε]	57942029	3998	6.9	6.69-7.12

[†] Sex-adjusted per 100,000 inhabitants/year White population in Monroe county, [γ] Age-and-sex adjusted rates of first hospital admissions, [¶] ICD-8 Danish version, [χ] Population: girls aged 15-25, calculated based on data from 2011, [η] Age-sex specific annual incidence rate, [ϑ] Population: girls aged 15-25, based on data from 2016, [Φ] Age- and- sex-adjusted rates per 100,000 woman-years, [δ] Average annual age-standardized hospital first recorded admission rates for anorexia nervosa per 100,000 woman-years in Oxford and West Berkshire (England), [ε] Average annual age-standardized hospital first recorded admission rates for anorexia nervosa per 100,000 woman-years in England.

Table S4. Incidence of anorexia nervosa per 100,000 woman-years in outpatient healthcare services.

Study	Country	Criteria	Ages	Period	N	n	Incidence	95% CI
Kendell et al. 1973 [41]	Scotland	No data	15-34	1960-69	250000	27	10.8	7.12-15.70
	England				170730	7	4.1	1.65-8.40
Cullberg & Engström-Lindberg, 1988 [36]	Stockholm	DSM-III	All	1984-85	38864	2	5.2	0.51-10.92
			16-24	1984-85	4651	2	43.0	5.22-155.67
Pagsberg & Wang, 1994 [45]	Denmark	ICD-10	10-24	1970-74	5128	2	7.8	0.95-28.18
				1975-79	5088	4	15.7	4.28-40.26
				1980-84	4940	3	12.1	2.50-35.50
				1985-89	4557	13	57.1	30.38-97.57
Lucas et al. 1999 [39]	USA	DSM-III-R	All†	1935-49	211761	31	15.0‡	9.30-20.60
				1950-59	184315	16	7.6‡	3.90-11.40
				1960-69	247660	34	12.8‡	8.50-17.20
				1970-79	295988	44	14.5‡	10.66-19.70
				1980-89	33492	68	22.9‡	17.30-28.60
			15-24	1935-49	45319	12	26.5	13.68-46.25
				1950-59	35698	11	30.8	15.38-55.13
				1960-69	47852	26	54.3	35.49-79.61
				1980-89	58268	44	75.5	54.87-101.37
Rooney et al. 1995 [37]	UK	DSM-III-TR	All	1991-92	267800	13	4.9	2.58-8.30

		DSM-III-TR	15-29	1991-92	62400	12	19.2	9.90-33.60
Turnbull et al. 1996 [38]	England & Wales	DSM-IV	All	1993	1264292	100	7.9	6.40-9.50
Hoek et al. 2005 [44]	Curaçao	DSM-IV	All	1995-98	320651	11	3.4	1.40-5.46
Currin et al. 2005 [40]	UK	General practice	All	2000	758945	65	8.6	6.61-10.92
van Son et al. 2006 [46]	Netherlands	DSM-IV	5-64	1985-89	373975	50	13.4	9.92-17.63
				1995-99	379880	57	15.0	11.36-19.44
			15-24	1985-89	63055	30	47.6	32.10-67.92
				1995-99	50133	33	65.8	45.31-92.44
Micali et al.2013 [47]	UK	ICD-10	10-49	2009	1302016	177	13.6	11.67-15.75
			15-29	2009	394442	135	34.2	28.70-40.51
Zerwas et al. 2015 [48]	Denmark	ICD-10	12-24	1995-12	1078214	775	71.8	66.91-77.12
Steinhausen & Jensen, 2015 [49]	Denmark	ICD-10	4-65	1995	2697854	267	9.9	8.75-11.16
				2000	3518518	380	10.8	9.74-11.94
				2005	4530000	453	10.0	9.10-10.96
				2010	496195	521	10.5	9.62-11.44
Smink et al. 2016 [50]	Netherlands	DSM-IV	5-64	2005-09	347764	41	11.8	8.50-16.00
			15-24	2005-09	40874	27	66.1	43.53-96.11
Tsai et al. 2018 [42]	Taiwan	ICD-9-CM	10-34	2001-2012	2548077	53	2.1	1.52-2.64
			15-24	2001-2012	979339	32	3.3	2.23-4.60
Mei-Chih et al. 2019 [43]	Taiwan	ICD-9-CM	10-49	2002-2013		1691	2.0	1.91-2.10
			15-29	2002-2013		1166	4.1	3.74-4.44
Reas & Rø, 2018 [5]	Norway	ICD-10	10-49	2010		947	36.9	33.10-39.70
				2011	2609187¶	995	38.1	34.80-41.60
				2012		1103	42.3	38.90-45.90

	2013		1082	41.5	38.10-45.10
	2014		984	37.7	34.50-41.10
	2015		980	37.6	34.40-40.90
	2016		1031	39.6	36.30-43.10
	2010		279	84.3	74.70-97.00
	2011		280	84.7	74.98-95.10
	2012		334	101.0	90.38-112.30
15-29	2013	3330969[g]	324	97.9	87.52-109.10
	2014		317	95.9	85.52-106.90
	2015		291	88.1	78.11-98.60
	2016		311	93.9	83.81-105.00

[†] Sex-adjusted per 100,000 inhabitants/year White population in Monroe county; [‡] Incidence per 100.000 person-years directly age adjusted to 1970. U.S.;Whites; [§] Average annual age-standardized hospital first recorded admission rates for anorexia nervosa per 100,000 people, [¶] overall female in 2016, [g] Female 15-29 in 2016.

Table S5. Incidence of anorexia nervosa per 100,000 woman-year in cohort studies.

Study	Country	Records	Criteria	Age	Period	N	n	Incidence	95% CI
Keski-Rakhnonen et al. 2007 [51]	Finland	Twin cohorts	DSM-IV	15-19	1985-89	2545	55	270.0	180.00-360.00
Ghaderi & Scott, 2001 [53]	Sweden	Cohort	DSM-IV	20-32	1997-99	1157	1	120.0	61.99-481.56
Lahortiga-Ramos et al. 2005† [54]	Spain	Cohort	EAT-40/DSM-IV	20-32	2004	2509	8	318.9	137.66-628.27

†Incidence in 18 months of follow-up.
